# Distinct Roles for Sialoside and Protein Receptors in Coronavirus Infection

**DOI:** 10.1128/mBio.02764-19

**Published:** 2020-02-11

**Authors:** Enya Qing, Michael Hantak, Stanley Perlman, Tom Gallagher

**Affiliations:** a Department of Microbiology and Immunology, Loyola University Chicago, Maywood, Illinois, USA; b Department of Microbiology and Immunology, University of Iowa, Iowa City, Iowa, USA; Vanderbilt University Medical Center

**Keywords:** coronavirus, virus entry, virus receptors, virus glycoproteins, sialic acids, membrane fusion

## Abstract

Coronaviruses (CoVs) are common human and animal pathogens that can transmit zoonotically and cause severe respiratory disease syndromes. CoV infection requires spike proteins, which bind viruses to host cell receptors and catalyze virus-cell membrane fusion. Several CoV strains have spike proteins with two receptor-binding domains, an S1A that engages host sialic acids and an S1B that recognizes host transmembrane proteins. As this bivalent binding may enable broad zoonotic CoV infection, we aimed to identify roles for each receptor in distinct infection stages. Focusing on two betacoronaviruses, murine JHM-CoV and human Middle East respiratory syndrome coronavirus (MERS-CoV), we found that virus particle binding to cells was mediated by sialic acids; however, the transmembrane protein receptors were required for a subsequent virus infection. These results favored a two-step process in which viruses first adhere to sialic acids and then require subsequent engagement with protein receptors during infectious cell entry. However, sialic acids sufficiently facilitated the later stages of virus spread through cell-cell membrane fusion, without requiring protein receptors. This virus spread in the absence of the prototype protein receptors was increased by adaptive S1A mutations. Overall, these findings reveal roles for sialic acids in virus-cell binding, viral spike protein-directed cell-cell fusion, and resultant spread of CoV infections.

## Abstract (precis)

CoVs can transmit from animals to humans to cause serious disease. This zoonotic transmission uses spike proteins, which bind CoVs to cells with two receptor-binding domains. Here, we identified the roles for the two binding processes in the CoV infection process. Binding to sialic acids promoted infection and also supported the intercellular expansion of CoV infections through syncytial development. Adaptive mutations in the sialic acid-binding spike domains increased the intercellular expansion process. These findings raise the possibility that the lectin-like properties of many CoVs contribute to facile zoonotic transmission and intercellular spread within infected organisms.

## INTRODUCTION

Coronaviruses (CoVs) are enveloped RNA viruses causing endemic infections in several mammalian and avian species. There are seven known human CoVs, each causing respiratory disease, together accounting for about one-third of common colds (1, 2). Those human CoVs that have only recently entered into humans from infected animals, severe acute respiratory syndrome coronavirus (SARS-CoV), Middle East respiratory syndrome coronavirus (MERS-CoV), and now 2019-nCoV, can cause severe, often fatal acute respiratory syndromes (3–10). CoV prevalence, facile zoonotic transmission, and potential to cause severe respiratory disease bring urgency to research aimed at discovering CoV infection mechanisms.

The mechanisms by which CoVs enter host cells, and subsequently spread intercellularly, may explain, in part, the remarkable expansion of these viruses into new hosts, including humans. Entry and spread are carried out by spike (S) glycoproteins (11, 12), homotrimeric, multidomain, integral membrane glycoproteins that protrude from virions and infected cells. The S proteins adhere viruses and infected cells to host cell receptors, and they subsequently function as catalysts of virus-cell membrane fusion, and in several CoV infection settings, cell-cell membrane fusion as well. With respect to the adherence step, it is notable that many CoV S proteins contain multiple distinct host cell receptor-binding domains (RBDs) (13–18). More than one RBD raises the question of whether RBDs operate independently, and if so, whether one RBD provides complete infection competence, while the other remains as an inactive, i.e., vestigial, domain. The MERS-CoV S proteins contain a N-terminal RBD (also known as the “S1A” domain [17]) that is structurally similar to galectins (17, 19, 20) and binds host cell sialic acids (21). MERS-CoV S proteins also have a C-terminal “S1B” RBD (17) that binds the protein receptor human dipeptidyl peptidase 4 (hDPP4) (22). Here, the S1B-hDPP4 binding is demonstrably essential for virus-cell entry (23, 24), while the biological significance of the S1A-sialic acid binding is currently unclear. In contrast, the murine hepatitis virus (MHV) S proteins also have galectin-like N-terminal S1A RBDs (13, 20, 25) but presumably lack sialic acid binding potential (19, 25), instead binding the protein receptor mCEACAM (mouse carcinoembryonic antigen-related cell adhesion molecule) (25). The MHV C-terminal S1B domains have no known receptor affinities, but they are known to control the thresholds for S-mediated membrane fusion activation (26–29). The fact that these two related beta-CoVs utilize distinct RBDs and host factors raises challenges in developing general models of CoV-cell binding, membrane fusion activation, and subsequent infection.

One hypothesis is that distinct RBDs operate at different CoV infection stages. Initial infection by virus particles likely requires durable adherence of virus particles to cell receptors. At later infection stages, CoVs produce S proteins in abundance, far exceeding the amounts that are incorporated into secreted progeny virus particles. These unincorporated S proteins accumulate on infected-cell plasma membranes where, depending on the CoV strain, they mediate cell-cell fusions, i.e., syncytial developments, which rapidly expand infection. Close cell-cell contacts may obviate the need for high-affinity adherence of S proteins to adjacent, uninfected cell surfaces. Conceivably, S1A RBDs with relatively low affinity for sialic acids might be sufficient to promote syncytial expansion of infection, without requiring high-affinity S1B-protein receptor interactions. This putative role for sialic acids has not been considered. This study provides results that implicate sialic acids in facilitating CoV infection and CoV cell-cell spread, bringing insights concerning CoV dissemination in nature.

## RESULTS

### JHM-CoV binds sialic acids on target cells.

Although the CoV strains comprising the MHVs have evolved protein (mCEACAM) binding determinants on their galectin-like S1A domains (20, 25), we considered it likely that some MHV CoV strains also retain sialic acid binding competence. This presumption came from studies of the JHM-CoV strain of MHV. JHM-CoV can infect mCEACAM knockout mice (30), and it can mediate cell-cell fusion of several mCEACAM-negative cell types (27, 31–35). Among the CoVs, only the JHM-CoV strain has these documented activities that are apparently independent of a protein receptor, making it a sensible virus to use in addressing the hypothesis that sialate- and protein receptor-binding activities coexist on CoV S1A domains.

**FIG 1:**
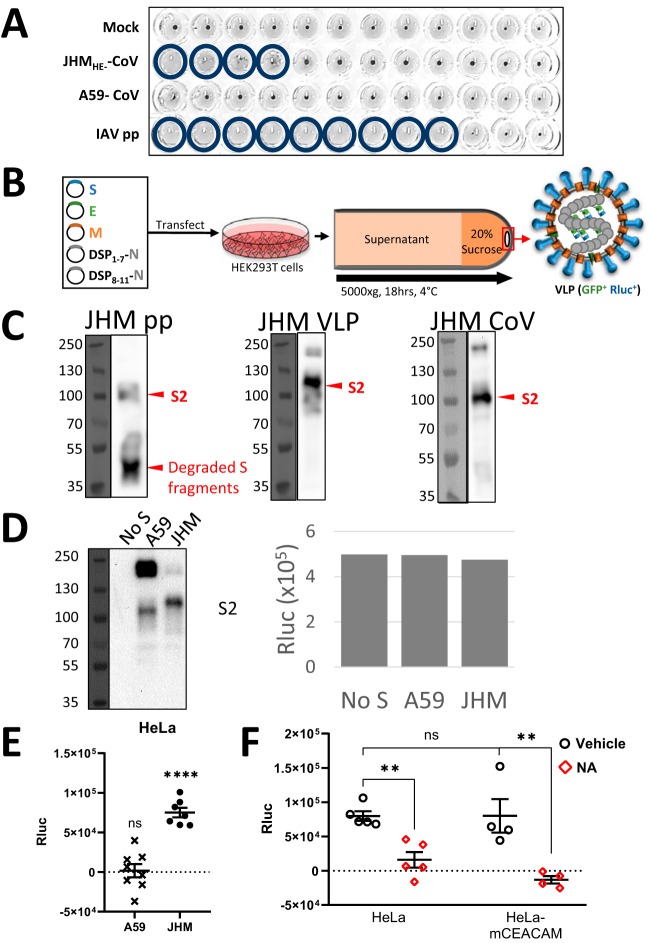
JHM S protein facilitates virus binding to sialic acids. (A) JHM_HE-_-CoV, A59-CoV, or IAV pps were serially twofold diluted, placed into V-bottom wells, and incubated for 2 h at 4°C. Human adult erythrocytes were then added to a final concentration of 0.25% (vol/vol). Hemagglutination was scored after 2 to 12 h at 4°C. The experiment was performed four times. (B) A59-CoV VLPs were collected from HEK293T cells expressing viral structural genes S, E, M, and DSP_1-7_-N or DSP_8-11_-N. VLPs were purified through 20% sucrose cushions and quantified by assessing Rluc levels. (C) Western blot detection of JHM S proteins in JHM pp, JHM VLP, and authentic JHM-CoV. (D, left) Western blot detection of A59 and JHM S proteins in VLPs. (Right) VLPs in each bar contained equal Rluc activity. (E) HeLa cells were formalin fixed, rinsed, and incubated for 2 h at 4°C with VLPs bearing A59 or JHM S. Rluc input multiplicities were equal for all VLP inoculations. Media were removed, cells were lysed, and cell-associated Rluc activities were quantified. Data are presented after subtracting background (“No S”) Rluc-positive (Rluc^+^) VLP levels, with each data point representing averages from independent experiments (*N* = 8 [A59] and 7 [JHM]; *n* = 4 technical replicates per experiment). Error bars present standard errors (SE) from the mean. Statistically significant deviations from background (“No S”) binding were assessed by unpaired Student’s *t* test and are indicated as follows: ns, not significant; *, *P* < 0.05; **, *P* < 0.01; ***, *P* < 0.001; ****, *P* < 0.0001. (F) HeLa or HeLa-mCEACAM cells were fixed and treated with vehicle (PBS) or neuraminidase (NA) for 3 h at 37°C. JHM-CoV VLPs were then added for 2 h at 4°C, cell-associated Rluc activities were quantified, and data are presented after subtracting background (“No S”) Rluc^+^ VLP levels. Error bars present standard errors (SE) from the mean. Statistically significant deviations were assessed by unpaired Student’s *t* test and are indicated as follows: ns, not significant; *, *P* < 0.05; **, *P* < 0.01; ***, *P* < 0.001; ****, *P* < 0.0001.

Sialic acid-binding viruses will agglutinate erythrocytes. To determine whether sialic acid binding has a critical role in JHM-CoV entry, we evaluated hemagglutination of human erythrocytes, using influenza A virus hemagglutinin (HA)-bearing pseudo particles (IAV pp) as positive controls. Because some JHM isolates can express and incorporate sialic acid-binding hemagglutinin-esterase (HE) proteins into virus particles (36, 37), we selected a recombinant JHM-CoV that lacks HE expression (JHM_HE−_-CoV) (see Fig. S1A in the supplemental material), so that any observable hemagglutination could be attributed to S proteins. Intriguingly, JHM_HE−_-CoV virus particles agglutinated human erythrocytes, while the related A59-CoV strain did not (Fig. 1A), even at particle-associated S-protein input levels slightly exceeding the corresponding JHM_HE−_-CoV (Fig. S1B). These findings suggested that JHM uniquely engages host sialic acids.

10.1128/mBio.02764-19.1 FIG S1 Western blot detection of virion proteins. (A) Purified recombinant JHM-CoV particles, normalized based on viral RNA abundance, were subjected to Western immunoblotting. Virion hemagglutinin (HE) proteins were detected using mouse anti-MHV-HE (α-MHV-HE) 5A11. (B) Purified A59-CoV and JHM_HE−_-CoV were subjected to Western immunoblotting. Virion spike (S) proteins were detected using mouse anti-MHV-S (α-MHV-S) (10G). The relative band intensities were used to normalize viral input in the hemagglutination assays. Download FIG S1, TIF file, 1.2 MB. Copyright © 2020 Qing et al. 2020 Qing et al. This content is distributed under the terms of the Creative Commons
Attribution 4.0 International license.

To validate the presumed JHM virus spike-host cell sialic acid interaction, we developed an MHV-based virus-like particle (VLP) system, in which VLPs contained reporter proteins that can be tracked during virus-cell binding and entry (Fig. 1B). In this system, VLPs were produced from 293T cells cotransfected with MHV structural genes S, E, M, and N (38–40). The N genes were modified by in-frame fusion with dual-split protein (DSP) encoding sequences, the two of which complement to form *Renilla* luciferase (Rluc) and green fluorescent protein (GFP) reporters. This allowed for the release of VLPs that were intrinsically Rluc and GFP positive.

We noted that MHV VLPs incorporated intact JHM spike proteins at levels that were similar to authentic viruses. This was in contrast to JHM spikes that were incorporated into vesicular stomatitis virus (VSV)-based pseudoparticles, most of which were degraded (Fig. 1C). These findings indicated that the VLPs structurally resemble authentic virus structures, at least with respect to spike stability and particle incorporation. As spike stability and multivalent presentation are frequently required to detect sialate interactions (21, 41), we considered the VLPs to be well-suited for receptor binding studies. Additionally, VLPs are equally produced either with or without incorporated S proteins (42), making it possible to use VLPs in quantifying S-protein-dependent virus-cell binding processes.

VLPs, either with or without JHM or A59 S proteins, were applied to mCEACAM-negative HeLa cells at equivalent (Rluc) multiplicities. Under these conditions, the S-protein levels on the A59 VLPs exceeded those of JHM VLPs (Fig. 1D). Yet, in agreement with the hemagglutinating effects of the viruses, the JHM S proteins increased VLP-HeLa cell binding above background levels, while the A59 S proteins did not (Fig. 1E). Notably, the JHM VLPs did not bind to HeLa cells that were pretreated with neuraminidase (Fig. 1F, left), suggesting that the VLP binding was dependent on cellular sialic acids.

Surprisingly, VLP binding to cells was not increased by expression of the MHV protein receptor mCEACAM, and furthermore, VLPs did not bind to mCEACAM-expressing cells that had been pretreated with neuraminidase (Fig. 1F, right). Together, these data suggest that, during MHV-JHM infection, the initial viral binding process is mediated by S-protein−sialic acid interactions, not by the protein receptor mCEACAM.

### Virus binding to sialic acids facilitates protein receptor-mediated entry.

**FIG 2:**
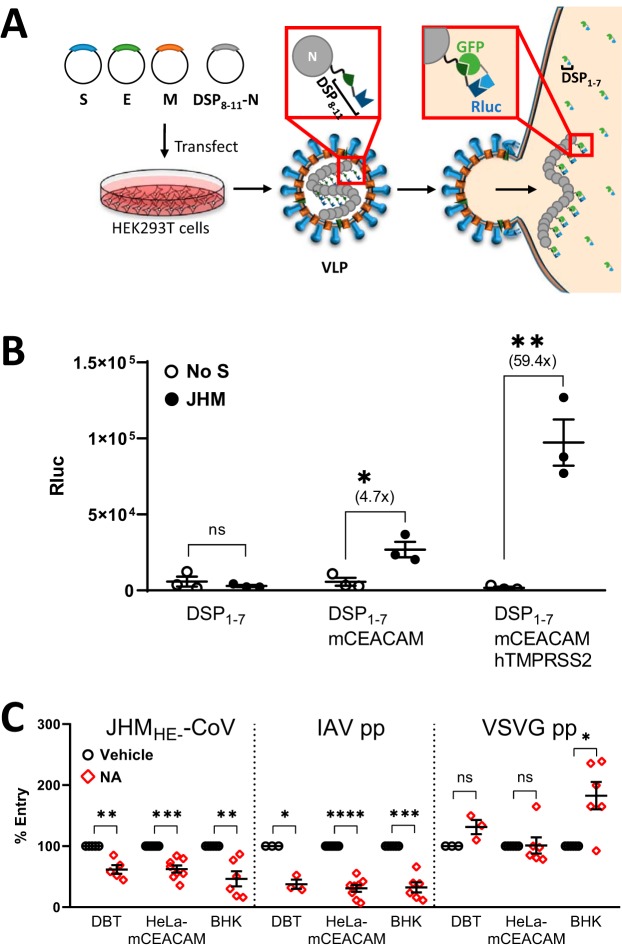
JHM S-protein binding to sialic acids facilitates cell entry. (A) VLPs containing DSP_8-11_-N were collected from transfected HEK293T cells and inoculated onto target cells expressing DSP_1-7_. Rluc signals arise after entry of DSP_8-11_-N into cells and subsequent complementation with DSP_1-7_. (B) HeLa target cells were transfected with DSP_1-7_, mCEACAM, and hTMPRSS2 as indicated. Two days posttransfection, DSP_8-11_-VLPs were inoculated for 6 h at 37°C, then cells were lysed, and Rluc activities were measured. (C) The indicated target cells were pretreated with vehicle or neuraminidase (NA) for 2 h at 37°C, followed by inoculation with the indicated virus particles for 2 h at 4°C. Unbound particles were removed, and after 16 h at 37°C, cells were lysed and Fluc was quantified. Data are presented as percent viral entry, normalized to vehicle controls. Statistical analyses were performed as described in the legend to Fig. 1.

We next aimed to identify the biological importance of the JHM spike-sialate interaction. We reasoned that this interaction could be sufficient for viral entry or may instead be an early step preceding viral engagement with protein (mCEACAM) receptors. To assess virus entry, we utilized VLPs containing N proteins fused to only one of the DSP fragments (DSP_8-11_), such that VLP entry into target cells containing DSP_1-7_ fragments could be measured by reporter complementation into active Rluc or GFP moieties (Fig. 2A). We compared VLP entries in HeLa and HeLa-mCEACAM cells and found that Rluc entry signals required mCEACAM presence (Fig. 2B). The entry signals were elevated by the presence of TMPRSS2 (Fig. 2B), a known viral fusion-triggering protease (11, 12, 43–48). This facilitating effect of TMPRSS2 supported the contention that the VLP-based entry assay faithfully reflected authentic CoV entry processes. In conjunction with VLP binding data, these findings suggest that JHM-CoV cell entry is a multistep process, with initial virus-sialate binding and subsequent mCEACAM receptor-driven entry.

We next determined whether virus binding to sialic acids elevates protein receptor-dependent virus entry. Here, authentic JHM_HE−_-CoV particles were bound for 2 h to DBT (murine), HeLa-mCEACAM (human), or BHK (hamster) cells, with or without prior cell exposure to neuraminidase. Infection was then measured by quantifying viral firefly luciferase (Fluc) reporter expression. The neuraminidase pretreatments decreased JHM_HE−_-CoV entry by 38% (DBT), 38% (HeLa-mCEACAM), and 54% (BHK) (Fig. 2C). After neuraminidase pretreatments, the cells were similarly resistant to IAV pp transduction, but not VSV G protein (VSV G)-containing pseudoparticle (VSVG pp) transduction (Fig. 2C). Partial resistance to IAV pp, a known sialic acid-requiring virus (49–51), is best explained by incomplete removal of sialic acids by neuraminidase. We infer that the partial neuraminidase resistance of CoV VLPs is similarly explained by incomplete sialic acid removal. These results support a model in which initial virus binding to sialic acids facilitates protein receptor-mediated entry.

### Sialic acid receptors promote JHM viral spread without requiring mCEACAM protein receptors.

**FIG 3:**
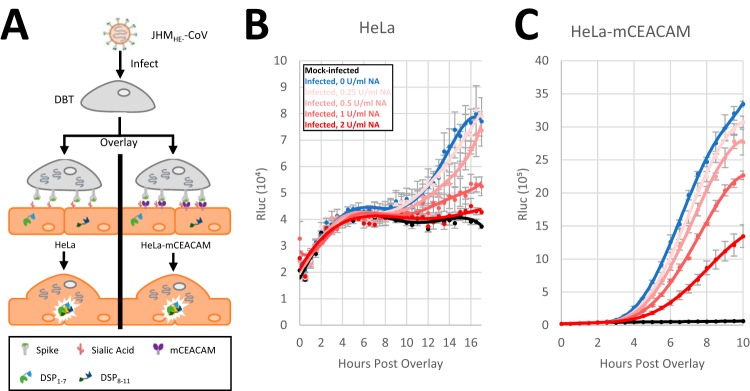
JHM-CoV cell-to-cell spread depends on host sialic acids. (A) Schematic for JHM S-protein-mediated cell-cell fusion measurements. JHM-CoV-infected DBT cells (multiplicity of infection [MOI] = 10; 5 h postinfection [hpi]) were added to target HeLa or HeLa-mCEACAM cells. The target cells were cocultures of cells expressing DSP_1-7_ and DSP_8-11_. JHM-CoV-infected cells fuse with multiple target cells, which enables DSP_1-7_−DSP_8-11_ complementation into active Rluc. (B and C) Quantification of Rluc over time in mCEACAM-negative (B) or mCEACAM-positive (C) target cells, in the presence of the indicated NA concentrations. Means (data points), SE (error bars), and the polynomial trend lines (*R*^2^ > 0.9) are shown. The results are representative of two biological repeats.

CoV infections spread through the canonical process of progeny virus release and reinfection, and also through fusion, i.e., syncytial spread, with uninfected cells. JHM S proteins can uniquely mediate syncytia in several conditions, even when mCEACAM protein receptors are absent (33, 35). To determine whether sialic acids operate independently of mCEACAM receptors in JHM cell-cell membrane fusion and syncytial development, we cultured JHM CoV-infected mouse (DBT) cells with mCEACAM-negative human (HeLa) cells that separately harbored either DSP_1-7_ or DSP_8-11_, such that HeLa cell-cell fusions would permit DSP complementation into active Rluc (Fig. 3A). Using live-cell Rluc substrate, the development of active Rluc was measured over time, and in the presence of increasing neuraminidase doses. The results (Fig. 3B) revealed neuraminidase dose-dependent reductions in JHM CoV-induced syncytia, up to 7.4-fold at the highest neuraminidase dose (2 U/ml). Similar, but less pronounced reductions were observed in parallel cultures of CEACAM-positive HeLa cells (Fig. 3C). Of note, the neuraminidase treatments significantly reduced cell-surface sialic acids, as revealed by adherence of fluorescent wheat germ agglutinin (WGA) to treated cells (Fig. S2). From these findings, we conclude that sialic acids can enable a CoV S-mediated cell-cell fusion process without requiring a prototypic proteinaceous receptor.

10.1128/mBio.02764-19.2 FIG S2 Immunofluorescence micrographs of target cells after NA treatments. HeLa or HeLa-mCEACAM cells were pretreated with NA at the indicated doses at 37°C. Following rinses, cell surface sialate levels were assessed by coincubating cells with Alexa Fluor 647-conjugated wheat germ agglutinin (WGA-Alexa647) (Molecular Probes) at 4°C. Following fixation and Hoechst staining, images were captured y using an EMCCD Cascade 2 camera and processed in Imaris 8.3.1. Images are representative of three technical repeats. Download FIG S2, TIF file, 4.5 MB. Copyright © 2020 Qing et al. 2020 Qing et al. This content is distributed under the terms of the Creative Commons
Attribution 4.0 International license.

### A JHM-CoV spike mutation increases mCEACAM-independent cell binding and membrane fusion.

**FIG 4:**
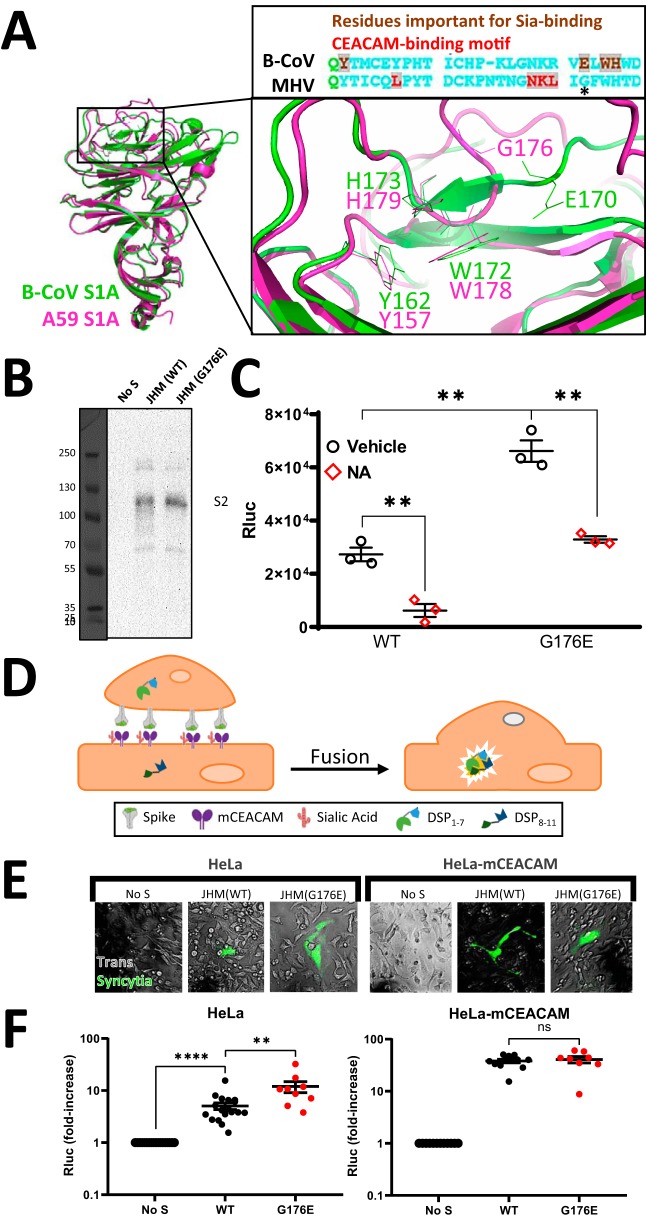
JHM S1A G176E mutation elevates target cell binding and mCEACAM-independent cell-cell fusion. (A) Structural superimposition and sequence alignment of B-CoV S1A (PDB accession no. 4H14, green) and A59 S1A (PDB accession no. 3JCL, magenta) using PyMol. Side chains in stick representation are included for residues of interest. (B) Western blot detection of VLP-associated S proteins. (C) VLPs lacking S proteins, or bearing JHM (WT) or JHM (G176E) S proteins, were applied to HeLa cells at equivalent Rluc levels. Experimental procedures, data acquisition, and processing were performed as described in the Fig. 1 legend. (D) Schematic for JHM S-protein-mediated cell-cell fusion measurements. GFP and Rluc signals arise only after S-expressing effector cells fuse with target cells, which enables DSP_1-7_−DSP_8-11_ complementation. (E) Live-cell images of the syncytia formed by S-expressing cells, without (left) or with (right) mCEACAM on target cells. Images were captured by an EMCCD Cascade 2 camera and processed in Imaris 8.3.1. (F) Quantification of syncytial developments. After imaging, cells were lysed and their Rluc activities werequantified. Data are presented as fold increases above background (“No S”) values. Statistical analyses were performed as described in the Fig. 1 legend.

To date, analyses of the multidomain CoV S proteins have revealed that the S1A domains interact with sialic acids (18, 19, 21, 41) and that most of the S1B domains bind to protein receptors (22, 52, 53). The exceptions are the MHV beta-CoVs, which have S1A domains that bind to protein (CEACAM) receptors (25). From these findings, we inferred that the JHM-CoV S1A domains contain a novel dual-receptor binding capability, able to bind both sialic acid and CEACAM receptors. Sialic acid-binding sites on beta-CoV S proteins were originally inferred from mutagenesis studies (19), and most recently, from structural resolution of S proteins in complex with sialosides (18, 54). Mutations in the inferred site did decrease sialoside binding (19, 41), even though they are distal from the structurally resolved sialoside binding grooves (18, 54). Here, we noted a mutation in the original inferred site. Among a stretch of four residues important for sialate-binding activity of bovine CoV (B-CoV) S1A, JHM differed only at residue 176, where there is a Gly on a divergent loop in place of the orthologous B-CoV Glu170 (Fig. 4A). To evaluate the importance of this divergence to sialic acid binding, we constructed a G176E mutant JHM-CoV S protein. G176E S-protein expression, proteolytic processing, and VLP incorporation were all comparable to the wild type (WT) (Fig. 4B). Notably, VLPs with G176E spikes bound more tightly to HeLa cells, and G176E spike-containing VLP binding was suppressed by neuraminidase pretreatment of the cells (Fig. 4C). To correlate this increased cell binding with membrane fusion, we compared the syncytial potency of JHM wild-type and G176E mutant S proteins. Cell-cell fusions were measured by DSP_1–7_−DSP_8–11_ complementation (Fig. 4D), visualized as GFP-positive syncytia (Fig. 4E), and quantified by measuring Rluc signals (Fig. 4F). Of note, when mCEACAM receptors were present, synyctia induced by wild type and mutant S proteins developed comparably, but in the absence of mCEACAM, the G176E spikes were significantly more robust in their syncytium-inducing activity (Fig. 4F). We concluded that a single point mutation in the JHM-CoV S1A domain increases the cell binding and membrane fusion activities of S proteins, independent of the prototype JHM protein receptor.

### MERS-CoV spikes mediate cell fusion without requiring hDPP4 protein receptors.

**FIG 5:**
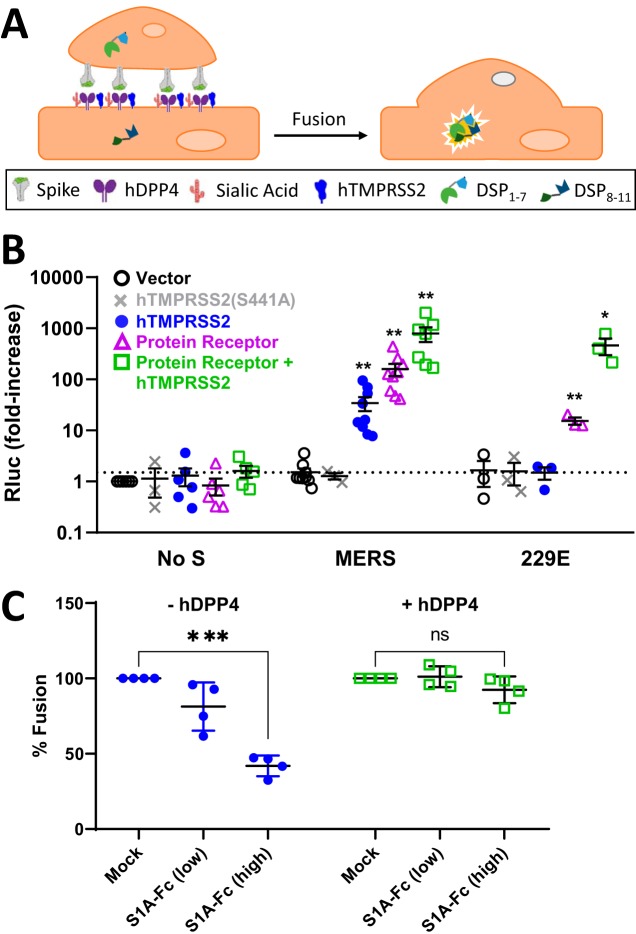
MERS S protein facilitates cell-cell fusion in the absence of hDPP4. (A) Schematic for MERS S-protein-mediated cell-cell fusion measurements. (B) Effector cells expressing the indicated S proteins were cocultured with target cells expressing hTMPRSS2 and/or protein receptors (hDPP4 for MERS, and hAPN for 229E). The dotted line represents background Rluc signals. (C) MERS S-expressing effector cells were mixed with hTMPRSS2 or hTMPRSS2/hDPP4 target cells in the presence of soluble MERS-S1A-Fc proteins. Data acquisition and processing were performed as described in the Fig. 1 legend.

MERS-CoV S1B domains bind to host protein (hDPP4) receptors (22–24), and MERS-CoV S1A domains bind to host sialic acids (21). This raised the question of whether MERS-CoV spikes can catalyze cell fusions after the binding of their S1A domains to cells, and independently of high-affinity protein receptors, similar to the JHM-CoV spikes. To address this question, MERS-CoV S-mediated cell-cell fusions were measured by DSP_1–7_−DSP_8–11_ complementation (Fig. 5A) and quantified by measuring Rluc signals (Fig. 5B). Of note, syncytial development mediated by MERS-CoV S proteins did not require hDPP4 but did require the S-protein-activating protease human TMPRSS2 (hTMPRSS2) (Fig. 5B). The catalytically inactive TMPRSS2(S441A) did not facilitate cell-cell fusion (Fig. 5B). The MERS S-mediated cell fusions contrasted with those generated by HCoV-229E S proteins, which do not bind sialic acids (21). 229E S-mediated fusions absolutely required target-cell human aminopeptidase N (hAPN) receptors (Fig. 5B). Thus, with sufficient cell-surface protease activities, the primary hDPP4 receptor was dispensable, conceivably because the secondary sialate-binding S1A RBDs can operate at a stage in the process.

To determine whether the MERS S1A domains operate in the cell fusion process, S1A-Fc proteins (Fig. S5) were added during the assays. Exogenous S1A proteins interfered with hDPP4-independent MERS S-mediated fusion but had no effect on fusion when hDPP4 was present (Fig. 5C). To determine whether sialic acids operate in the cell fusion process, neuraminidase was added during the assays. Exogenous neuraminidase reduced hDPP4-independent MERS-S-mediated fusion but had no effect on the cell fusion when hDPP4 was present (Fig. S3). These results further suggest that either of the two S domains, S1A or S1B, can tether S proteins to target cells for subsequent cell-cell fusions, with distinct host receptors for each domain.

10.1128/mBio.02764-19.3 FIG S3 Target cell sialic acids affect MERS S-mediated, hDPP4-independent, cell-cell fusion. (A) Schematic for MERS S-protein-mediated cell-cell fusion measurements. Rluc signals arise only after S-expressing effector cells fuse with target cells, which enables DSP_1-7_−DSP_8-11_ complementation. Syncytial development was quantified by measuring Rluc signals over time. (B and C) The kinetics of syncytial developments of hDPP4-negative (B) or hDPP4-positive (C) target cells, in the presence of the indicated NA concentrations. Means (data points), SE (error bars), and the polynomial trend lines (*R*^2^ > 0.9) are shown. The results are representative of two biological repeats. Download FIG S3, TIF file, 2.0 MB. Copyright © 2020 Qing et al. 2020 Qing et al. This content is distributed under the terms of the Creative Commons
Attribution 4.0 International license.

### An analogous MERS-CoV spike mutation similarly increases hDPP4-independent cell binding and membrane fusion.

**FIG 6:**
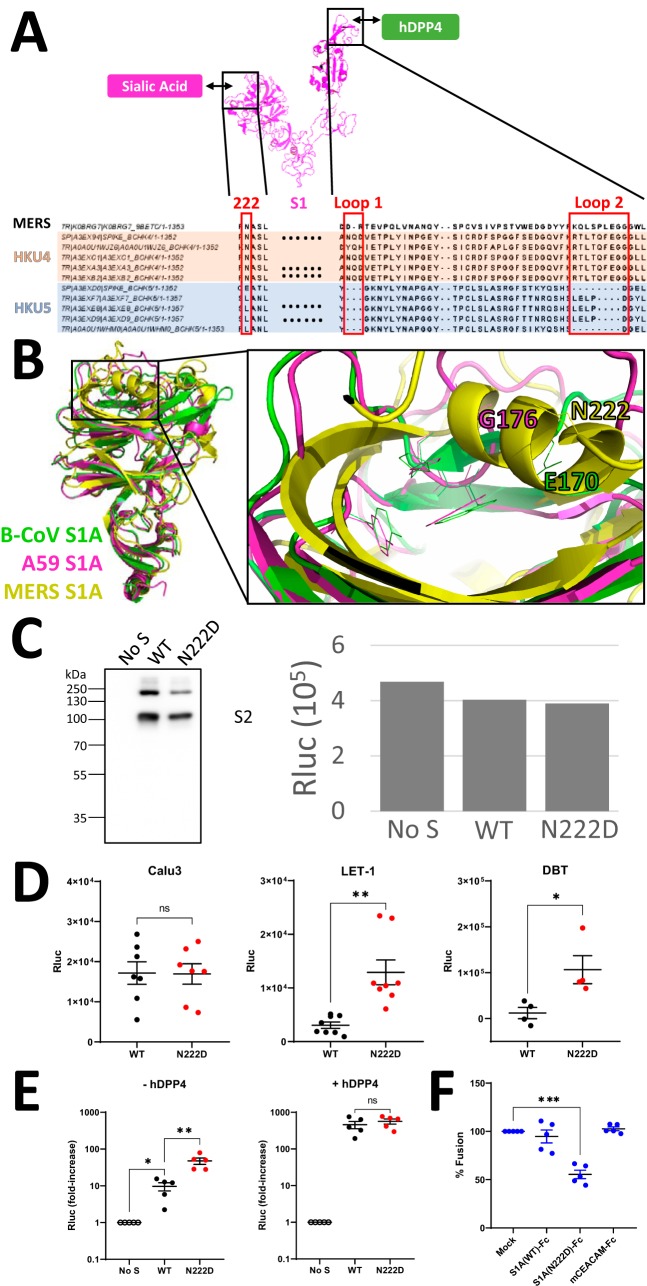
The N222D mutation on MERS-CoV elevates target cell binding and hDPP4-independent cell-cell fusion. (A) Comparison of selected MERS S sequences relative to bat MERS-like HKU4 and HKU5. MERS S residue N222, as well as hDPP4-binding loops 1 and 2, are delineated by red boxes. MERS S1 structure is from PDB accession no. 5X59. (B) Structural superimposition of B-CoV S1A (PDB accession no. 4H14, green), A59 S1A (PDB accession no. 3JCL, magenta), and MERS S1A (PDB accession no. 5X4R, yellow) using PyMol. MERS S1A residue N222 (stick representation) is located proximally to B-CoV S1A residue E170. (C) MERS VLP binding characterization. MERS-VLPs with equivalent Rluc activities (right) were processed for Western blotting to image S-protein abundance and integrity (left). (D) VLPs were added at equivalent Rluc input multiplicities to human Calu3 or murine LET-1 cells. After 2 h at 4°C, cell-associated Rluc activities were quantified, and data were presented after subtracting background (“No S”) Rluc^+^ VLP levels. (E) Effector cells expressing the indicated S proteins were cocultured with target cells expressing hTMPRSS2 and/or hDPP4. (F) MERS S-expressing effector cells were mixed with hTMPRSS2-expressing target cells, in the presence of the indicated Fc proteins. All Fc proteins were at 10 μM concentration throughout the coculture period. Experimental procedures, data acquisition, and processing were performed as described in the Fig. 1 legend.

We next determined whether analogous changes in S1A of the related MERS-CoV also modified viral attachment. The S1A interaction with sialic acids has biological significance, as antibodies against S1A protect mice from lethal MERS-CoV infections (55), and S1A acquires adaptive mutations in humans (56) and in mouse models of human MERS-CoV lung infection (57, 58). In mice, this selectivity was at Asn222 (57, 58), a known glycan addition site (17, 59, 60), and a documented hypervariable residue, evident in a group of MERS-CoV-related HKU4 bat viruses but absent in the HKU5 bat virus groups (Fig. 6A). Remarkably, in the context of CoV S1A structure, the Asn222 position overlaps closely with B-CoV residue Glu170 and MHV-CoV residue Gly176 (Fig. 6B). We constructed MERS VLPs to determine whether this Asn222 change impacted cell binding. In initial tests, we constructed MERS-CoV VLPs and evaluated their sialate binding properties. Human red blood cells (RBCs) were agglutinated by MERS VLPs, while neuraminidase-treated RBCs were not (Fig. S4), indicating that VLPs do bind sialosides. We then constructed wild-type and N222D VLPs containing internal Rluc and compared their Rluc and S-protein levels relative to VLPs lacking S proteins (Fig. 6C). VLPs were applied to human lung-derived Calu3 cells, which express the human DPP4 receptor for MERS-CoV, and to mouse lung-derived LET-1 and mouse brain-derived DBT cells, neither of which express human DPP4 and thus do not bind MERS-CoV via a protein receptor. VLP binding was assessed by quantifying cell-associated Rluc. Asn222 change did not change VLP binding to human Calu3 cells but clearly enhanced VLP binding to murine DBT and LET-1 cells (Fig. 6D).

10.1128/mBio.02764-19.4 FIG S4 MERS VLPs agglutinate human erythrocytes. (A) The indicated IAV pps or MERS VLPs were placed in V-bottom wells and incubated for 1 h at room temperature. The VLPs (No S or MERS S) were present at equivalent levels, as measured by their internal Rluc contents. Prewarmed (37°C) human adult erythrocytes, either with or without prior NA treatment, were added to a final concentration of 0.25% (vol/vol). Hemagglutination was scored after 2 to 12 h at room temperature. The experiment was performed two times. (B) MERS VLPs (No S or MERS S) were serially twofold diluted, placed into V-bottom wells, and incubated for 1 h at room temperature. Prewarmed (37°C) human adult erythrocytes were added to a final concentration of 0.25% (vol/vol). Hemagglutination was scored after 2 to 12 h at room temperature. The experiment was performed two times. Download FIG S4, TIF file, 2.3 MB. Copyright © 2020 Qing et al. 2020 Qing et al. This content is distributed under the terms of the Creative Commons
Attribution 4.0 International license.

We next considered whether this adaptation for increased cell binding had consequences in MERS S-mediated cell-cell membrane fusion, similar to the JHM-CoV G176E mutant. We found that the N222D mutant S proteins did indeed exhibit enhanced cell fusion, relative to wild-type S proteins (Fig. 6E), but this was only observed by eliminating hDPP4 (Fig. 6E), which removes the dominant S1B-protein receptor interaction and demands S1A utilization. To further probe the relationships between S1A-cell binding and cell fusion, we determined whether N222D S1A-Fc proteins would suppress cell fusion more effectively than WT S1A-Fc. Indeed, we found that exogenously added N222D S1A-Fc proteins suppressed cell fusions more potently than the corresponding wild-type S1A-Fc did (Fig. 6F and Fig. S5), further reinforcing the direct relationship between S1A binding to cells and resultant cell-cell fusion. Altogether, these findings support the hypothesis that MERS-CoV and JHM-CoV can acquire increased cell binding through S1A mutation, which consequently allows for more robust cell-cell fusion capability. Both of these CoVs can procure these cell binding and cell fusion properties through similarly localized S1A mutations.

10.1128/mBio.02764-19.5 FIG S5 Western blot detection of purified Fc-tagged proteins. Protein concentration was assessed using Nanodrop (Thermo Fisher), and human IgG1 (hIgG1) (Sigma) was loaded as a quantification control. Download FIG S5, TIF file, 0.8 MB. Copyright © 2020 Qing et al. 2020 Qing et al. This content is distributed under the terms of the Creative Commons
Attribution 4.0 International license.

## DISCUSSION

Viruses frequently begin infection by attaching to cellular sialic acids (61). Such viruses include several CoVs, which attach to sialates via spike S1A domains (11, 12), at low affinity (18), but relatively high multivalent avidity (21, 41). Atomic resolution structures of 9-O-acetylated sialic acid in complex with the human OC43-CoV S1A domain (18) as well as several sialosides in complex with the MERS-CoV S1A (54) have revealed architectures of CoV-sialate binding sites. Yet even with this detailed understanding, it is not clear whether sialic acids confer susceptibility to CoV infection on their own or whether proteinaceous CoV receptors are also required. In considering this question, we first focused on the murine MHV JHM-CoV strain. This strain can infect cells and mice that lack the proteinaceous MHV receptor, mCEACAM1a (27, 30, 32–35, 62, 63). This strain is also unusually sensitive to proteolytic activation of spike-mediated membrane fusion (46), and therefore, we hypothesized that a low-affinity cell binding event, conceivably to cellular sialoglycans, might be sufficient for subsequent JHM-CoV protease-triggered fusion activation and cell entry.

**FIG 7:**
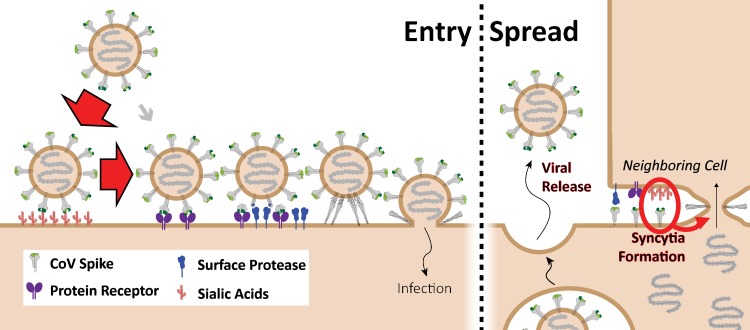
Sialic acid and protein receptor binding events during CoV infection. In the Entry panel, CoV S proteins mediate weak interactions with abundant host surface sialates, keeping viruses concentrated on cells yet potentially diffusible across plasma membranes. S proteins subsequently engage protein receptors and are proteolytically activated into membrane fusion-inducing conformations. In the Spread panel, canonical virus release is concomitant with cell-cell fusion. Cell-cell fusion involves S binding to sialic acids and does not require protein receptors, allowing infection to spread beyond the restricted distributions of protein receptors.

We found that JHM-CoV binding to cells depended on host sialic acids. This binding accounted for initial virus attachment to cells, which facilitated subsequent virus engagement with proteinaceous CEACAM receptors. Later in the infection cycle, JHM-CoV spikes facilitated cell fusions and further virus dissemination in a sialic acid-dependent manner. MERS-CoV spike proteins could similarly fuse cells together without requiring prototype protein (hDPP4) receptors, presumably by utilizing sialoside receptors to engage neighboring cells. These findings, summarized in Fig. 7, suggest that CoV-cell entry and intercellular spread involve the lectin-like activities of spike proteins during both virus-cell attachment and infected-cell expansion into syncytia.

While sialic acids affected virus-cell binding, infection by JHM-CoV and cell entry by MERS-CoV VLPs required the proteinaceous receptors mCEACAM and hDPP4, respectively. Therefore, one possibility is that multivalent CoV-sialate interactions are not sufficient to hold viruses throughout a virus particle-cell membrane fusion process and that sialic acids may instead only tether viruses, transiently, onto host cell surfaces, as they diffuse two-dimensionally and then bind more stably to high-affinity protein receptors. Yet, while insufficient for the initial infection, sialic acid binding may advance the spike-mediated cell-cell membrane fusions that are observed in several CoV infection processes. In cell-cell fusion, spikes on infected-cell surfaces are steadily directed at uninfected (target) cells. They are not liable to diffuse away as cell-bound viruses would, yet they still may require attachment to target cell sialates in order to effect membrane fusion. In support of this contention, we found correlations between sialic acid abundance and membrane fusion activity. This was observed for both JHM- and MERS-CoV spikes, and notably, both the JHM- and MERS-CoV spike-directed cell fusions took place independently of any proteinaceous receptors. These correlations suggest that sialic acids on uninfected cells can arrange spikes such that they will then operate cooperatively to pull opposing plasma membranes into coalescence.

This differential utilization of sialoside and protein receptors raises questions about CoV evolution. The CoV S1A domains fold into a galectin-like structure (14, 15, 17–19, 25, 64), and several CoV S1A domains demonstrably bind carbohydrate ligands (18, 21, 41, 54), but prior to this report, the MHV-CoV S1A domains were considered to bind only protein (mCEACAM) receptors (25). However, Peng et al. (19) proposed that ancestral MHV-CoVs bound carbohydrates, with adaptive evolution generating the present-day CEACAM-binding sites. This proposal infers the existence, past or present, of evolutionary intermediates capable of binding both mCEACAM and sialic acid. The MHV JHM-CoV strain meets the criteria for such an intermediate—a virus in which a single domain retains two “receptor” binding activities, one for carbohydrate and the other for protein. Conceivably, these two receptor sites interact, such that increased affinity for one ligand reduces affinity for the other. Mutants with relatively high affinity for sialic acid may bind poorly to mCEACAM, while strains lacking sialic acid binding may demonstrate strong mCEACAM affinity. An inverse relationship would be consistent with the study of Peng et al., who proposed that acquisition of a CEACAM-binding site concomitantly destroyed a sialate site (19). Yet perhaps more intriguing relationships are between CoV S and hemagglutinin-esterase (HE) proteins, both of which bind sialosides (65, 66). Esterase activities within HE proteins deacetylate sialosides, and it has been documented, in HKU1-CoV infections, that HE activity destroys an S-specific sialoside receptor, keeping viruses detached from cells and allowing for virus dissemination (67). Several MHV strains, including JHM, express a viral HE, which binds and deacetylates 4-O-acetyl sialiate (68). Similar to HKU-1-CoV HE, MHV HE could destroy MHV sialoside receptors and facilitate dissemination. MHV strain A59 did not bind sialic acids, and it does not retain an intact HE open reading frame (36, 37), while strain JHM did bind sialic acids and does express HE, suggesting that HE is necessary for those CoVs with relatively high S-protein affinity for sialosides. MERS-CoV binds sialic acids, albeit weakly (21), and increases in its affinity for sialates may be limited by the absence of a MERS-CoV HE gene.

This study revealed S-protein mutations that may endow CoVs with expanded tropisms, beyond that determined by prototype proteinaceous CoV receptors. The JHM-CoV mutation G176E, engineered to reflect sialate-utilizing B-CoV, increased viral S-protein binding to cells, as well as S-protein-mediated membrane fusion, independently of mCEACAM. The MERS-CoV mutation N222D, an adaptation for virus growth in mouse lungs (58), operated remarkably similar to the JHM-CoV G176E change, with mutant S proteins showing increased hDPP4-independent cell binding and cell fusion. Our findings fit with the hypothesis that viruses with these mutations bind relatively tightly to sialoside receptors. However, it is clear that the changes are not present in currently identified sialoside-binding sites (18, 54), which raises alternative hypotheses that include mutation-induced allosteric restructuring of sialoside-binding sites. Alternatively, the mutations may restructure distinct receptor- or coreceptor-binding sites, such as presumed binding sites for orthologous CEACAMs on MHV S proteins (69), or sites for CEACAM5 (70) or GRP78 (71) on MERS-CoV S proteins.

Finally, this report illuminates understanding of CoV pathogenesis. Among the CoVs, the JHM-CoV strain is known for causing lethal brain infection, even in mice that lack the principal MHV receptor, CEACAM1a (30). JHM spike was identified to be the major contributor to this phenotype (30), and JHM-CoV, but not the related A59-CoV, spread interneuronally both *in vivo* and within *in vitro* cultures of central nervous system (CNS)-derived cells (89, 90). Notably, neural cell membranes are known for their abundant sialic acid content (72). These findings, combined with evidence that cell-to-cell syncytial spread correlates with pathogenesis in several infection models (30, 62, 63, 73–75), prompts a hypothesis that JHM-CoV sialic acid binding potential accounts for an interneuronal syncytial spread that is rapidly lethal. A prediction is that variants of JHM-CoV exhibiting enhanced sialic acid affinity will have unusually high neurovirulence. Similarly, the MERS-CoV strain causes lethal pneumonia, and here it is significant that antibodies specific for the MERS-CoV S1A domains both neutralize the virus and reduce infection and pathogenesis in a mouse MERS-CoV model system (55, 59, 76). Conceivably, these antibodies interfere with sialic acid binding, reducing expansion of MERS-CoV that may take place via cell-cell fusion. Variants of MERS-CoV with enhanced cell binding may be useful in assessing the *in vivo* significance of the findings presented in this report.

## MATERIALS AND METHODS

### Cells.

HEK293T (ATCC), HeLa (ATCC), and HeLa-mCEACAM (77, 78) cells were maintained in DMEM−10% FBS medium (Dulbecco’s modified Eagle medium [DMEM] containing 10 mM HEPES, 100 nM sodium pyruvate, 0.1 mM nonessential amino acids, 100 U/ml penicillin G, and 100 μg/ml streptomycin, and supplemented with 10% fetal bovine serum [FBS] [Atlanta Biologicals]). BHK-21 cells (ATCC) were maintained in DMEM−5% FBS medium. LET-1 cells (BEI Resources) (79) were maintained in DMEM−10% FBS medium lacking HEPES, sodium pyruvate and nonessential amino acids. Calu3 cells (ATCC) were maintained in MEM−20% FBS medium (minimum essential medium [MEM] supplemented with 20% FBS, 100 U/ml penicillin G, and 100 μg/ml streptomycin). DBT cells (80, 81) were maintained in MEM−5% FBS medium (MEM supplemented with 5% FBS, 10% tryptose-phosphate broth, 26.8 mM sodium bicarbonate, 2 mM l-glutamine, 100 U/ml penicillin G, and 100 μg/ml streptomycin). All cell lines were cultured in a 5% CO_2_ incubator at 37°C.

### Viruses.

Recombinant MHV strains JHM.SD (82) and A59 (83), both containing a firefly luciferase (Fluc) reporter between the viral E (envelope) and M (matrix) genes, were grown in DBT cells. Media were collected at 24 to 48 h postinfection. JHM_HE−_ arose during laboratory passaging of JHM.SD.

### Virus-like particles.

CoV virus-like particles (VLPs) were constructed by cotransfection with equimolar amounts of plasmids encoding CoV S, E (envelope), M (matrix), and N (nucleocapsid). Coding sequences for A59-CoV S, E, M, and N genes are presented in GenBank accession no. AY910861.1; for JHM-CoV, GenBank accession no. AC_000192.1; and for MERS-CoV (EMC 2012 strain [84], GenBank accession no. JX869059.2, where only the S gene is codon optimized [24]). The A59/JHM-CoV and the MERS-CoV genes were inserted into pCAGGS and pcDNA3.1 expression vector plasmids, respectively. Recombinant pCAGGS-DSP_1-7_-N and pCAGGS-DSP_8-11_-N were constructed by fusing the DSP_1-7_ or DSP_8-11_ coding sequences (pDSP_1-7_ and pDSP_8-11_ [85, 86] provided by Zene Matsuda [University of Tokyo]), followed by a 2× SGGS linker, to the 5′ ends of the coding sequences for the N genes.

Expression plasmids (1 μg) were complexed with polyethylenimine (PEI) (Polysciences) (6 μg) in 0.2 ml of Opti-MEM (Life Technologies) for 15 min at room temperature and then added dropwise to 5 × 10^5^ HEK293T cells in 1 ml of DMEM−10% FBS. For spikeless VLPs, S expression plasmids were replaced with empty vector plasmid DNAs. Six hours later, the cells were replenished with fresh DMEM−10% FBS. Media were collected at 48 h posttransfection.

### Pseudoviruses.

Pseudovirus particles (pps) were constructed from a vesicular stomatitis virus (VSV) platform as described in reference 87. Briefly, HEK293T cells were transfected with viral glycoprotein expression plasmids, including pHEF-VSV G-Indiana (BEI Resources), pcDNA3.1-HA5-QH, and pcDNA3.1-PR8 NA1 (obtained from Lijun Rong, University of Illinois—Chicago). Twenty-four hours later, the cells were inoculated for 2 h with VSVdeltaG/Junin GP-luciferase (87, 88). The cells were rinsed twice with FBS-free DMEM medium and replenished with DMEM−10% FBS. Media were collected after a 48-h incubation period.

### Particle concentration.

Media containing viruses, pseudovirus particles, and VLPs were clarified by differential centrifugation (300 × *g*, 4°C, 10 min; 3,000 × *g*, 4°C, 10 min). Particles were concentrated from clarified media by overnight centrifugation (SW28, 6500 rpm, 4°C, 18 h) through a cushion comprised of 20% (wt/wt) sucrose in FBS-free DMEM. The resulting pellets were resuspended in FBS-free DMEM, and the resulting 100× concentrated particle stocks were stored at –80°C.

### Hemagglutination assay.

Serial twofold dilutions of 100× concentrated virus and pseudovirus particle stocks were prepared using FBS-free DMEM as diluent. Diluted samples were placed into V-bottom 96-well plates and incubated for 2 h at 4°C. Washed adult human erythrocytes (0.5%) (in phosphate-buffered saline [PBS]) were then added, and scoring was performed after 2- to 12-h incubation at 4°C.

### Neuraminidase treatments.

Adherent, confluent cells in 24-well plates were rinsed once with PBS before fixation with 3.7% paraformaldehyde in 0.159 M piperazine-*N*,*N*′-bis(2-ethanesulfonic acid) (PIPES) for 30 min at room temperature. Following three PBS rinses, cells were treated with vehicle or neuraminidase (1 U/ml) (catalog no. N2876; Sigma) in neuraminidase buffer (200 Mm sodium acetate [NaOAc], 2 mM CaCl_2_ [pH 5.0]) for 3 h at 37°C. The cells were then rinsed three times with PBS and then blocked with PBS containing 2% FCS (PBS + 2% FCS) for 30 min at room temperature, and then used in virus binding assays.

For live-cell assays, adherent, confluent cells in 24- or 96-well plates were pretreated with vehicle or neuraminidase (1 U/ml) (catalog no. N2876; Sigma) in FBS-free DMEM for 2 h at 37°C. The cells were rinsed three times with PBS and then used in virus transduction assays.

### VLP binding assay.

VLPs used for binding assays were prepared by cotransfection with plasmids encoding CoV S, E (envelope), M (matrix), and DSP_1-7_-N + DSP_8-11_-N. The resulting VLPs contained complemented DSP Rluc-positive interiors. To quantify VLP-associated Rluc, VLPs were serially diluted into passive lysis buffer (PLB) (catalog no. E194A; Promega) and introduced into opaque microplate wells (50 μl per well). *Renilla* luciferase substrate (1.1 M NaCl, 2.2 mM Na_2_EDTA, 0.22 M KH_2_PO_4_, 1.3 mM NaN_3_, 0.44 mg/ml bovine serum albumin, 2.5 μM coelenterazine [pH 5]) was added (100 μl per well), and luminescence was read using a Veritas microplate luminometer (Turner BioSystems, Sunnyvale, CA).

VLPs were incubated for 2 h at 4°C to adherent, confluent cells, either with or without prior neuraminidase treatments. Incubations were in FBS-free DMEM and at known Rluc VLP/cell ratios. After incubation, unbound VLPs were removed, and cells were rinsed variably with PBS. To measure cell-bound VLPs, adherent cells were dissolved into PLB, and Rluc activity was quantified.

### VLP entry assay.

VLPs used for entry assays were prepared by cotransfection with plasmids encoding CoV S, E (envelope), M (matrix), and DSP_8-11_-N. The resulting VLPs contained DSP fragments in their interiors. To quantify VLP-associated DSP levels, VLPs were mixed with excess DSP_1-7_, in passive lysis buffer, for 30 min at 37°C, to allow for DSP complementation. The excess DSP_1-7_ was obtained from HEK293 cells overexpressing these fragments. After postlysis complementation, samples were introduced into opaque microplate wells and luminescence readings were used to infer DSP_8-11_ levels.

VLPs in FBS-free DMEM were inoculated onto target cells that were transfected 2 days earlier with pDSP_1-7_. Indicated experiments included cotransfection of target cells with pDSP_1-7_ and pCAGGS-TMPRSS2_FLAG_ (44). For VLP inoculations, input multiplicities were normalized to VLP DSP_8-11_ levels. After 6 h at 37°C, the cells were rinsed three times with PBS and dissolved in passive lysis buffer, and Rluc activities were quantified.

### Virus and pseudovirus particle entry assays.

Virus particles were incubated with cells, either with or without prior neuraminidase treatments, for 2 h at 4°C. Cells were then rinsed three times with PBS and replenished with FBS-containing DMEM/MEM. After 16 h at 37°C, cells were dissolved in lysis buffer (25 mM Tris-phosphate [pH 7.8], 2 mM dithiothreitol [DTT], 2 mM 1,2-diaminocyclohexane-*N*,*N*,*N*′-tetraacetic acid, 10% [vol/vol] glycerol, 1% Triton X-100), and Fluc levels were quantified with Fluc substrate (1 mM d-luciferin, 3 mM ATP, 15 mM MgSO_4_·H_2_O, 30 mM HEPES [pH 7.8]) and a Veritas microplate luminometer.

### Cell-cell fusion assay.

Effector and target cells were prepared by introducing expression plasmids, using the PEI transfection method. Effector cells were cotransfected with pDSP_1-7_ and the indicated S-expressing plasmids. S-expressing plasmids included pcDNA3.1-229E S_C9_ (GenBank accession no. AB691763.1, obtained from Fang Li, University of Minnesota). Control effector cells received pDSP_1-7_ and empty vector plasmids. Target cells were cotransfected with pDSP_8-11_ and the indicated S receptor-expressing plasmids. These plasmids included pCMV6-Entry-hDPP4_FLAG_ (GenBank accession no. NM_001935; purchased from OriGene) and pcDNA3.1-hAPN (GenBank accession no. M22324; obtained from Fang Li). Indicated experiments included additional cotransfection of target cells with pCAGGS-TMPRSS2_FLAG_ (44).

At 6 h posttransfection, effector and target cells were rinsed with PBS, lifted with 0.05% trypsin, and mixed at 1:1 ratios. The cocultures were incubated for 2 to 18 h at 37°C. Fused cells were visualized microscopically as green fluorescent protein-positive (GFP^+^) cells, and extents of cell-cell fusion were quantified as Rluc activities present in PLB cell lysates. Samples were chilled and maintained at 4°C during Rluc quantifications to eliminate DSP postlysis complementation. In time course experiments, EnduRen substrate (Promega E6482) was used in place of coelenterazine.

### Fc constructs.

pCEP4-mCEACAM-Fc was constructed previously (31). Splice overlap extension PCR was used to insert an MreI restriction site and GSGGGG linker codons between the mCEACAM (codons 1 to 142) and the human IgG1 splice donor site. Using this modified construct, the mCEACAM coding region was removed and replaced with MERS-S1A (codons 1 to 357), MERS S1A (N222D), and MERS S1B (codons 1 to 24 from human CD5 [hCD5] signal, followed by MERS S codons 358 to 588).

HEK293T cells were transfected using the PEI method and then incubated in FBS-free DMEM containing 2% (wt/vol) Cell Boost 5 (HyClone). Supernatants were collected on days 4 and 7 and clarified by sequential centrifugation (300 × *g*, 4°C, 10 min; 4,500 × *g*, 4°C, 10 min). The Fc-tagged proteins were then purified using HiTrap protein A high-performance columns (GE Healthcare) according to the manufacturer’s instruction. The resulting purified proteins were quantified spectrophotometrically and stored at –20°C until use.

### Western blotting.

Proteins in sodium dodecyl sulfate (SDS) solubilizer (0.0625 M Tris·HCl [pH 6.8], 10% glycerol, 0.01% bromophenol blue, 2% [wt/vol] SDS, with 2% 2-mercaptoethanol] were heated at 95°C for 5 min, electrophoresed through 8% (wt/vol) polyacrylamide-SDS gels, transferred to nitrocellulose membranes (Bio-Rad), and incubated with mouse monoclonal anti-MHV-HR2 10G (obtained from Fumihiro Taguchi, Nippon Veterinary and Life Sciences, Tokyo, Japan), mouse anti-C9 (EMD Millipore), mouse monoclonal anti-MHV HE, clone 5A11, or goat anti-human IgG (sc-2453; Santa Cruz Biotechnologies). After incubation with appropriate horseradish peroxidase (HRP)-tagged secondary antibodies and chemiluminescent substrate (Thermo Fisher), the blots were imaged and processed with a FluorChem E (Protein Simple).

### Microscopy and image acquisition.

Live-cell images were captured in a z series on an electron-multiplied charge-coupled-device digital camera (EMCCD Cascade 2; Photometrics) and deconvolved using SoftWoRx. Identical conditions were applied to all acquisitions. Deconvolved images were analyzed by an identical algorithm in Imaris 8.3.1 (Bitplane).

### Statistical analysis.

Unless stated otherwise, all experiments were repeated independently at least three times. Each data point graphed represents the mean of an independent repeat (*N*), generated from three or four technical repetitions (*n* = 3 or 4). Statistical comparisons were made by unpaired Student’s *t* test. Error bars indicate the standard errors of the data. *P* values less than 0.05 were considered statistically significant.
