# Supplementary figures and images for: Distinct Roles for Sialoside and Protein Receptors in Coronavirus Infection
Source: mBio. 2020 Feb 11;11(1):e02764-19. doi: 10.1128/mBio.02764-19 (PMC7018658; doi:10.1128/mBio.02764-19)

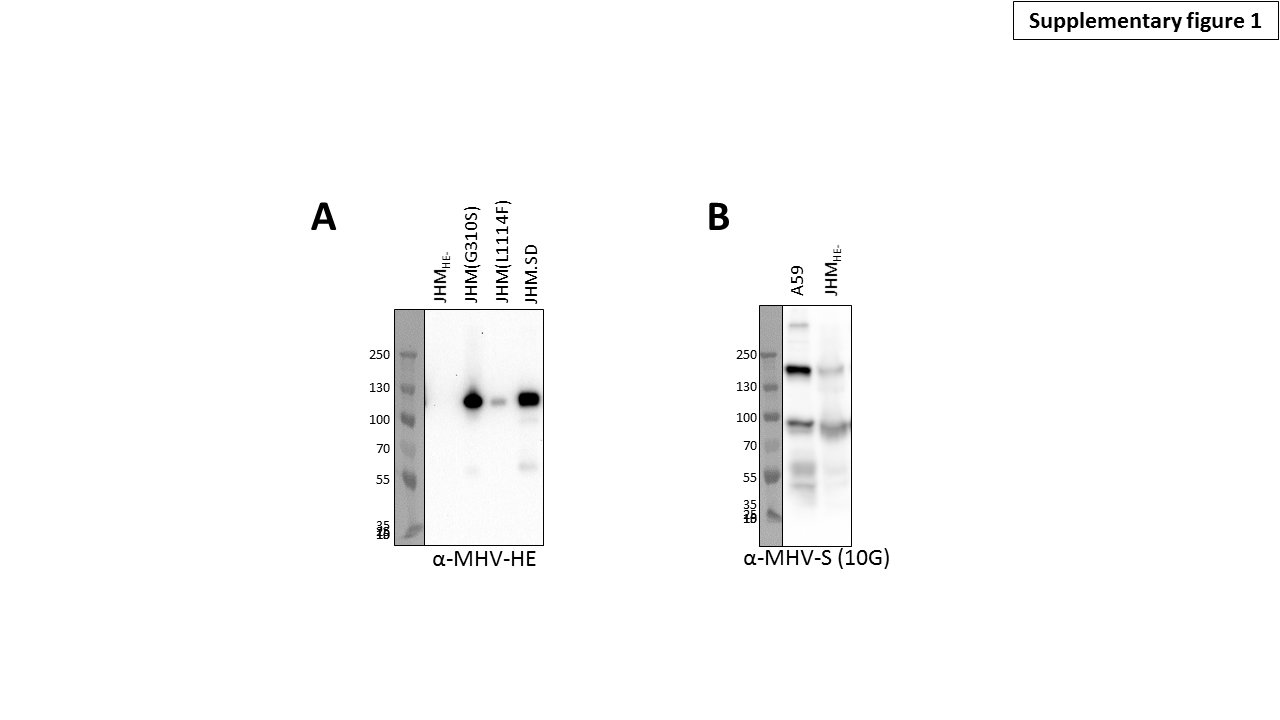

Supplement: FIG S1 [file mBio.02764-19-sf001.tif]

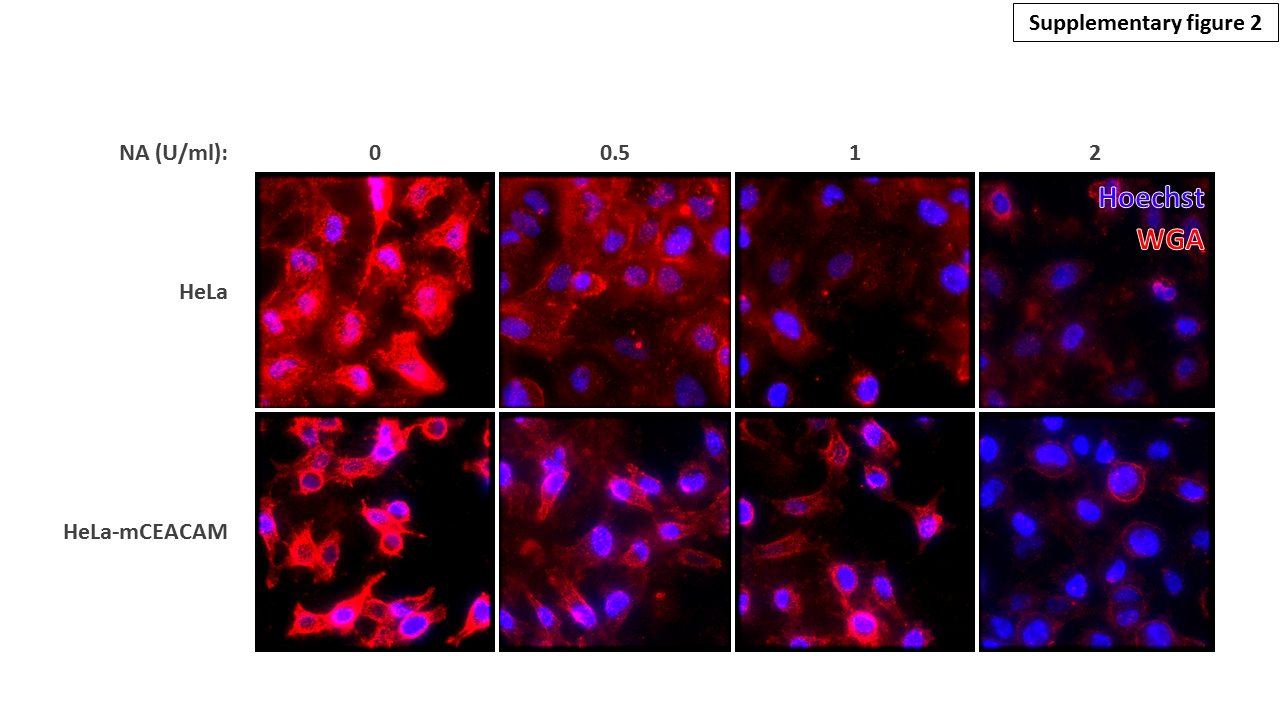

Supplement: FIG S2 [file mBio.02764-19-sf002.tif]

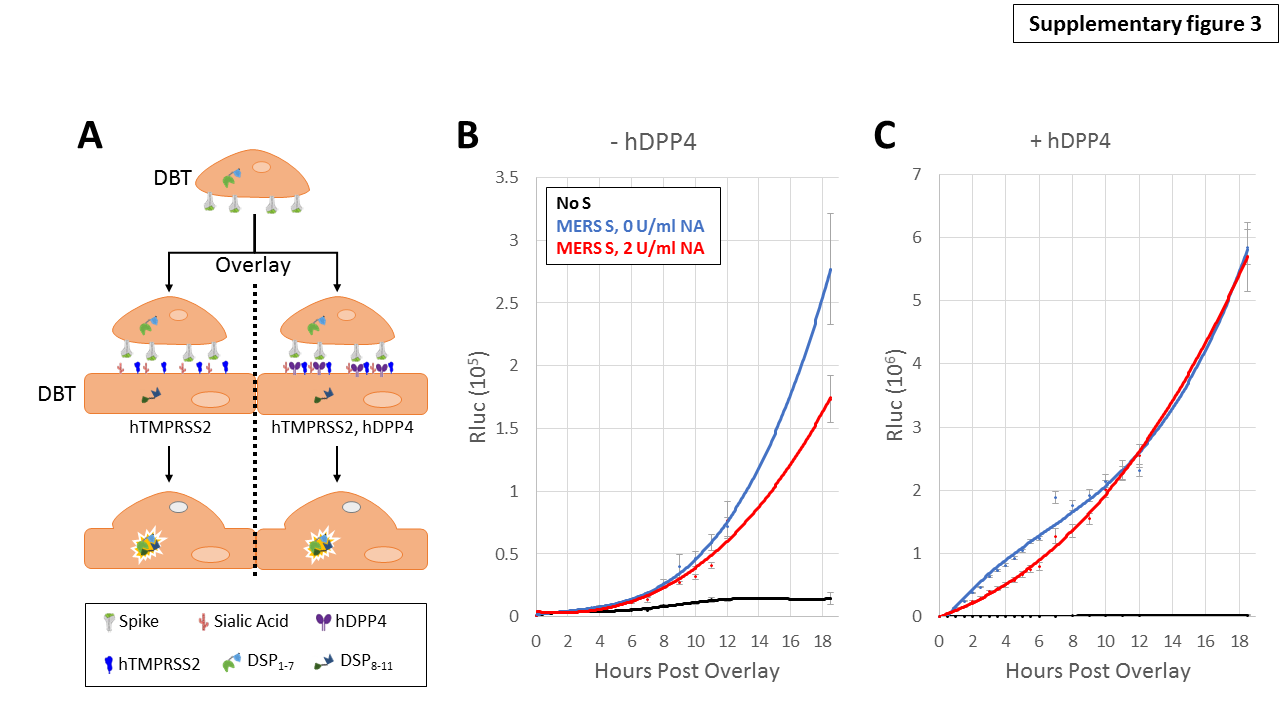

Supplement: FIG S3 [file mBio.02764-19-sf003.tif]

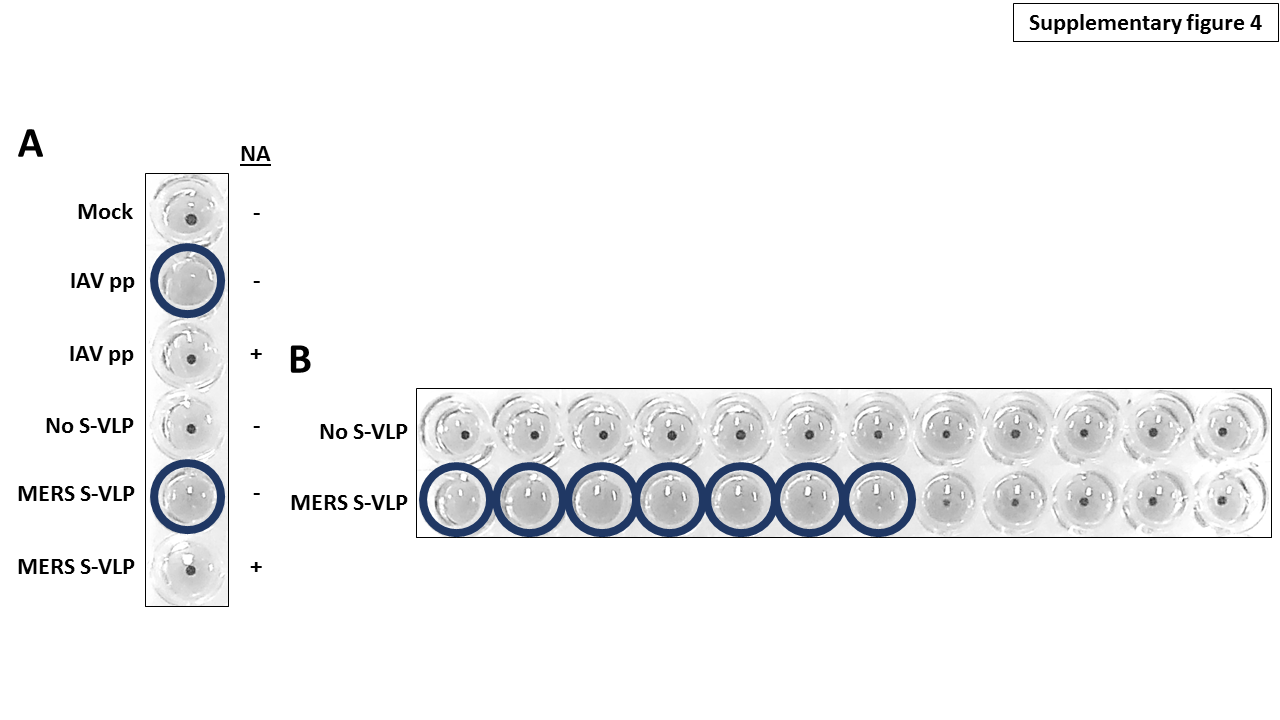

Supplement: FIG S4 [file mBio.02764-19-sf004.tif]

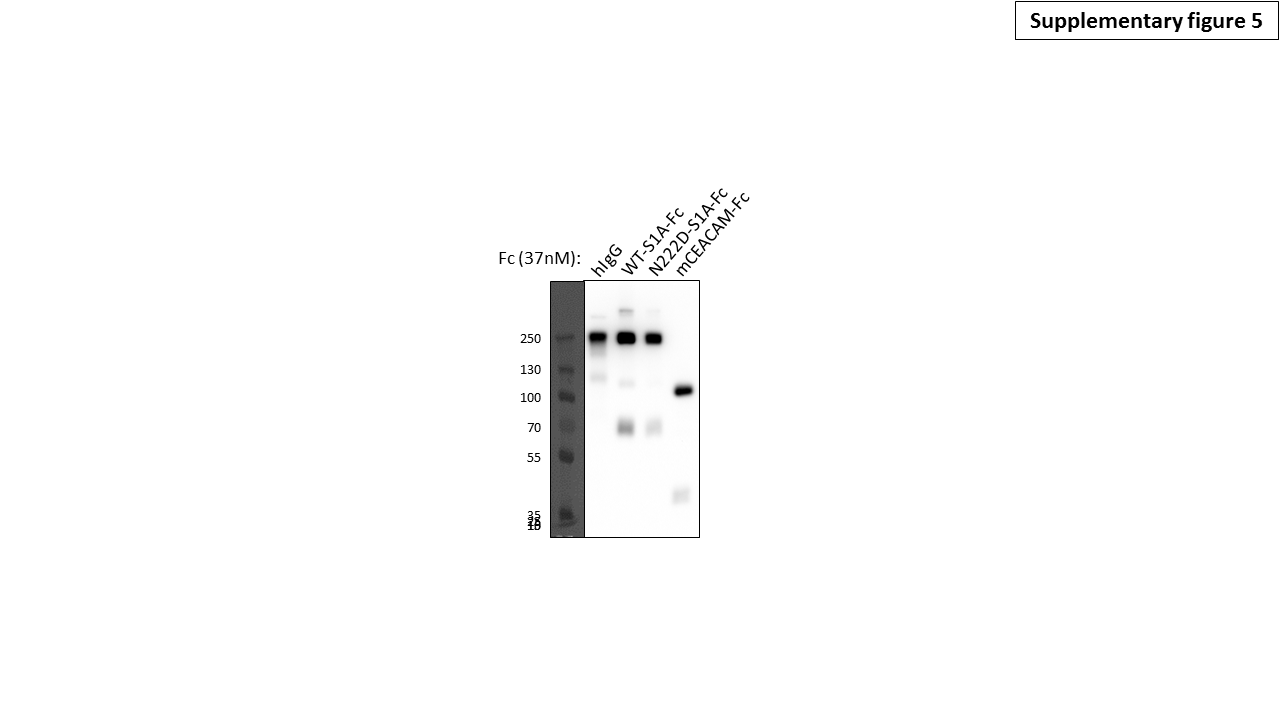

Supplement: FIG S5 [file mBio.02764-19-sf005.tif]
